# Posterolateral Corner Injury Associated with a Schatzker Type 2 Tibial Plateau Fracture

**DOI:** 10.1155/2015/527428

**Published:** 2015-10-27

**Authors:** Boris A. Zelle, James R. Heaberlin, Matthew C. Murray

**Affiliations:** Department of Orthopaedic Surgery, University of Texas Health Science Center at San Antonio, San Antonio, TX 78209, USA

## Abstract

Isolated posterolateral corner (PLC) injuries are rarely seen with tibial plateau fractures and can be missed during the initial assessment. The objective of this paper is to present a case of a Schatzker type 2 tibial plateau fracture with associated isolated PLC injury and give a discussion on physical exam, diagnostic studies, and treatment options. A twenty-five-year-old female sustained a concomitant Schatzker type 2 fracture and PLC injury. Magnetic Resonance Imaging showed an isolated PLC disruption. Open reduction-internal fixation was performed with subsequent PLC repair. At sixteen months postoperatively, the patient had full range of motion and strength of her knee and no signs of laxity. This case emphasizes the importance of physical exam and appropriate imaging modalities in order to diagnose and treat this significant injury in a prompt fashion. In this case, surgical fracture fixation and subsequent repair of the PLC provided a good clinical outcome.

## 1. Introduction

Soft tissue injuries associated with tibial plateau fractures are frequently seen in the clinical setting [1–5]. The posterolateral corner is frequently injured in tibial plateau fractures. However, it is more commonly injured in conjunction with the posterior cruciate ligament or multiple ligamentous injuries [6]. Isolated posterolateral corner (PLC) injuries appear less common, in particular in conjunction with Schatzker type 2 tibial plateau fractures. These injuries to the PLC may easily be missed during the initial assessment of a patient. Without proper recognition and treatment of this significant ligamentous injury, significant chronic pain, chronic posterolateral instability, and osteoarthritis may occur. The objective of this paper is to present a case of a Schatzker type 2 tibial plateau fracture with concomitant isolated posterolateral corner (PLC) injury and report the physical exam findings, imaging modalities, and treatment protocol.

## 2. Case Presentation

**Figure 1:**
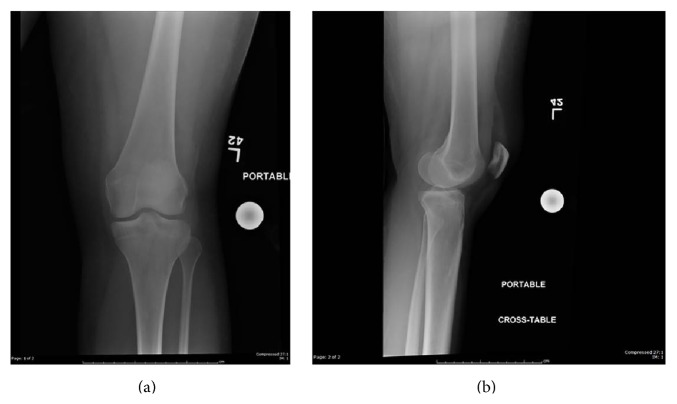
AP and lateral plain radiographs of the left knee showing a nondisplaced, vertically oriented fracture of the lateral tibial condyle.

**Figure 2:**
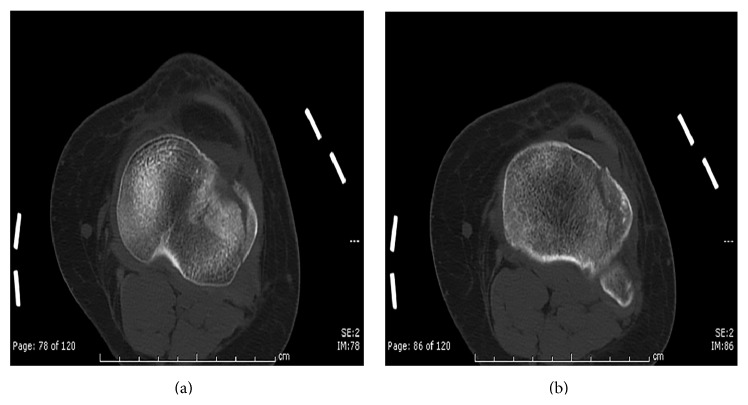
Initial CT scans of left tibia showing fracture of lateral tibial condyle.

**Figure 3:**
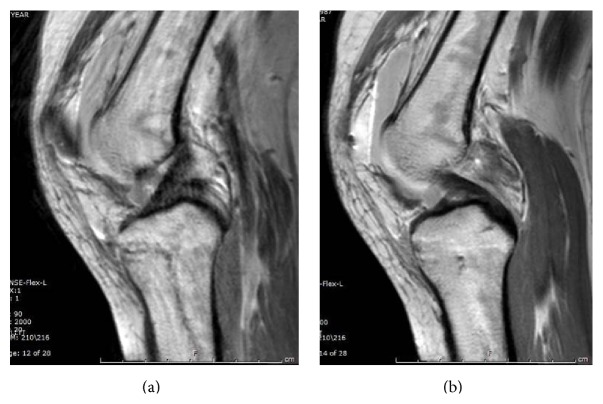
Initial sagittal MRI scans showing intact anterior cruciate ligament (a) and posterior cruciate ligament (b).

**Figure 4:**
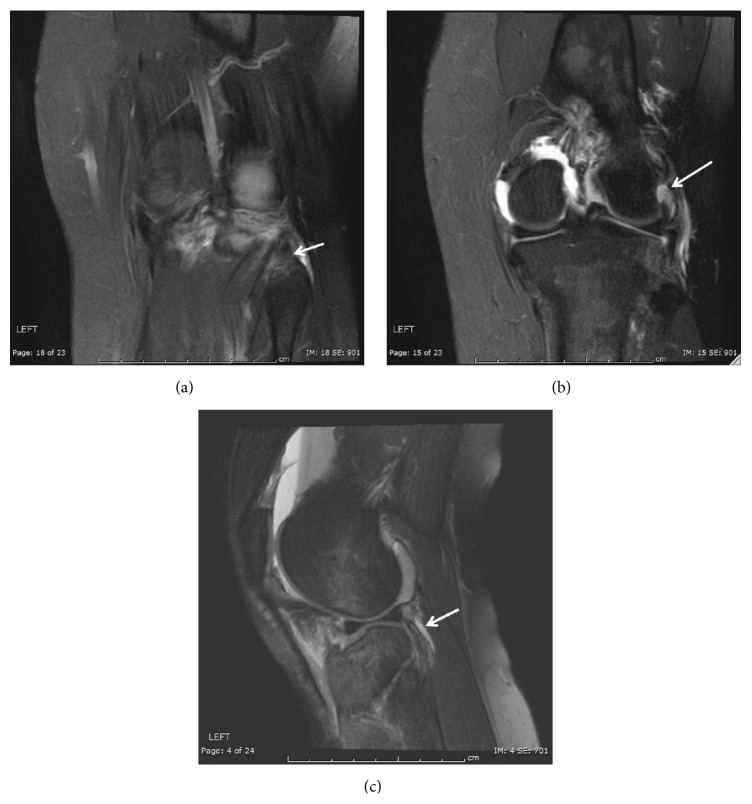
MRI of left knee showing popliteofibular ligament tear and edema in fibular styloid (arrow, (a)), attenuated LCL (arrow, (b)), and popliteus tendon signal abnormality suggesting interstitial tearing (arrow, (c)).

A twenty-five-year-old female was injured after falling from the back bumper of a moving truck. Her past medical history was significant for unspecified anemia and her surgical history included four previous cesarean sections. The patient presented to the emergency department of our level 1 trauma center and was evaluated by the orthopaedic trauma service and the general surgery trauma service. She was found to have an isolated injury to the left knee. Plain radiographs and computer tomography (CT) scans of the knee showed a lateral split depression type fracture of the tibial plateau (Schatzker type 2), mostly in the anterior portion with a vertical split (Figures 1 and 2). Upon application of a knee brace in the emergency department, obvious posterolateral instability was noted. A detailed ligamentous exam in the emergency department was deferred due to the acute injury and the patient's significant discomfort. Based on the physical exam findings, Magnetic Resonance Imaging (MRI) of the knee was indicated. The results of the MRI showed no injury to the anterior cruciate ligament, posterior cruciate ligament, medial collateral ligament, or meniscal damage (Figures 3(a) and 3(b)). However, the MRI demonstrated a concomitant injury to the posterolateral corner (Figures 4(a)–4(c)).

The patient was taken to the operating room on the first day after her injury. Open reduction and internal fixation was performed through a standard lateral approach to the proximal tibia including a submeniscal arthrotomy. Surgical fixation was achieved using a precontoured lateral proximal tibia plate by the manufacturer Smith and Nephew. After the fracture fixation, a detailed ligamentous exam was performed. This physical examination showed joint laxity to varus stress, 1+ at zero degrees and 2+ at thirty degrees. In addition, she had a positive tibial dial test at thirty degrees. These findings confirmed the diagnosis of posterolateral knee instability and established the indication for a PLC repair. A formal lateral dissection was performed and the peroneal nerve was protected and identified. The LCL had completely avulsed off the fibular head but remained firmly attached to the femur. The biceps femoris was partially torn and had sheared from the fibular head as well. Two suture anchors (1.5 mm Biomet Juggerknots, Warsaw, IN) were placed on the fibular at the respective insertions of the LCL and biceps. Direct repair of each was performed passing the suture in a Krackow fashion, giving good apposition of the ligament and tendon back to the fibula. The popliteus tendon appeared to be slightly stretched but in continuity and surgical repair was not deemed necessary. Afterwards, the knee was stable to varus stressing at 0 and 30 degrees. The incision was closed and the patient was admitted. After an uncomplicated postoperative hospital course, the patient was discharged to home with a knee brace locked in extension and non-weight-bearing instructions. At the two-week follow-up visit, the knee brace was unlocked and range of motion exercises were initiated. The patient was kept non-weight-bearing to the injured lower extremity for a total of 12 weeks.

**Figure 5:**
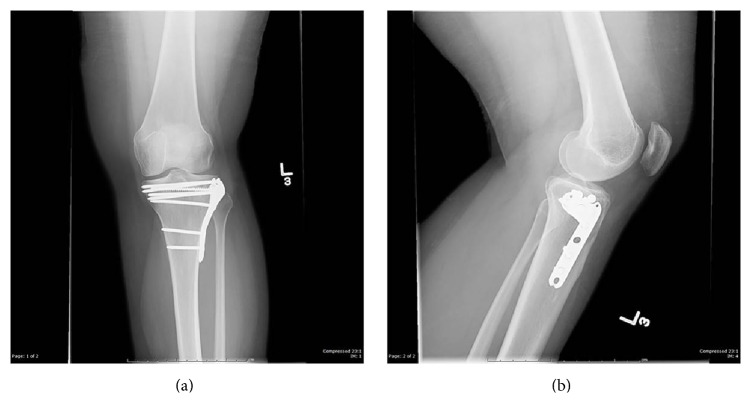
AP and lateral plain radiographs at sixteen months postoperatively.

The patient was last seen in the orthopaedic trauma clinic sixteen months after the operation when she returned to clinic for a follow-up appointment. On physical exam, she had 5/5 strength in her leg, had full range of motion from 0 to 140 degrees, and had no signs of joint laxity with anterior, posterior, valgus, or varus stress. Plain radiographs of the knee were taken at her sixteen-month postoperative visit (Figures 5(a) and 5(b)).

## 3. Discussion

The high incidence of soft tissue injury in association with tibial plateau fractures has been well established [1–5]. In one study, Gardner et al. [1] showed posterolateral corner (PLC) injuries were present in 68% of their patients with operative tibial plateau fractures. However, isolated PLC injuries are uncommon and are more commonly associated in addition to an injury to the posterior cruciate ligament or multiple ligamentous injuries [6]. If the injury is missed and left untreated, PLC injuries can lead to significant morbidity with chronic pain, chronic posterolateral rotator instability, and osteoarthritis [7–9]. Since prompt recognition and repair of the PLC within three weeks of injury has been suggested to have the best outcomes associated with repairs [10], we emphasize the importance of actively assessing for these injuries in patients with tibial plateau fractures.

The PLC is designed to resist varus stress, external rotation of the tibia, and posterior translocation of the tibia. The dial test is used frequently to assess the PLC by comparing it to the contralateral knee. Bae et al. [11] showed in their study that the dial test was helpful in the diagnosis of PLC injuries that had at least three structures damaged or PLC injuries with concomitant posterior cruciate ligament (PCL) injury. However, the study also showed if less than three structures are injured in isolated PLC injuries, the dial test can miss the diagnosis. In our case, two structures in the PLC were injured, the biceps femoris hamstring tendon and the LCL. However, our exam clearly showed a positive dial test. In addition to the dial test, varus laxity with the knee flexed at zero degrees and thirty degrees will also show injury to the PLC.

Plain radiographs of the knee can give valuable information such as soft tissue swelling and the position of the tibial condyle to the femur and fibula. Radiographic findings suggestive of an injury to the posterolateral corner include widening of the lateral joint space and a wide array of fracture patterns including tibial plateau fractures [6]. In an effort to help centers with limited MRI availability to have a guide for which soft tissue injuries are more commonly associated with plain radiograph measurements, Gardner et al. [12] reported Schatzker II fractures with 5 mm of depression or widening were more often associated with soft tissue injuries. These authors, however, acknowledged the MRI as the preferred imaging in conjunction with plain radiographs in diagnosis of soft tissue injury.

MRI has proven to be a useful tool in recognition of these soft tissue injuries in association with tibial plateau fractures. It allows for visualization of the soft tissues which have been injured throughout the knee and provides reliable and accurate data which can be used for preoperative planning [1, 13]. Yacoubian et al. [14] showed that MRI changed management in 23% of cases while Holt et al. [15] showed it changed the classification of the fracture in 47.6% and their management in 19% of their cases.

The use of arthroscopy as an intraoperative tool in the management of tibial plateau fractures remains controversial. Arthroscopy may allow for diagnosis and management of soft tissue injuries associated with tibial plateau fractures which may not be fully recognized through submeniscal arthrotomies and clinical examination [16]. However, there remains the potential risk of fluid extravasation and compartment syndrome as a potentially devastating complication.

## 4. Conclusion

Isolated PLC injury associated with a Schatzker type 2 fracture is a rare combination of injuries. Physical exam showed varus laxity and a positive dial test, with no signs of instability of other soft tissues. On MRI, soft tissue injury to the PLC was seen and operative fixation of the tibial plateau fracture and subsequent repair of the PLC were performed. To the best of our knowledge, there is little literature on the association of isolated PLC injuries with Schatzker type 2 tibial plateau fractures. For this reason, an associated isolated injury to the PLC can be easily missed during the assessment. This case report emphasizes the importance of preoperative and intraoperative physical examinations and appropriate use of MRI imaging if indicated by the clinical examination. Early diagnosis of this associated soft tissue injury may have a significant impact on the surgical treatment plan and patient outcomes.
